# Complete loss of the DNAJB6 G/F domain and novel missense mutations cause distal-onset DNAJB6 myopathy

**DOI:** 10.1186/s40478-015-0224-0

**Published:** 2015-07-25

**Authors:** Alessandra Ruggieri, Francesco Brancati, Simona Zanotti, Lorenzo Maggi, Maria Barbara Pasanisi, Simona Saredi, Chiara Terracciano, Carlo Antozzi, Maria Rosaria D′Apice, Federica Sangiuolo, Giuseppe Novelli, Christian R. Marshall, Stephen W. Scherer, Lucia Morandi, Luca Federici, Roberto Massa, Marina Mora, Berge A. Minassian

**Affiliations:** Neuromuscular Disease and Immunology Unit, Fondazione IRCCS Istituto Neurologico “C. Besta”, Via Celoria 11, 20133 Milan, Italy; The Centre for Applied Genomics and Program in Genetics and Genome Biology, The Hospital for Sick Children and University of Toronto, Toronto, Canada; Department of Molecular Genetics and the McLaughlin Centre, University of Toronto, Toronto, ON Canada; Department of Paediatrics (Neurology) and Program in Genetics and Genome Biology, The Hospital for Sick Children and University of Toronto, Room 6535, 555 University Ave, Toronto, ON M5G 1X8 Canada; Medical Genetics Unit, Policlinico Tor Vergata University Hospital, Rome, Italy; Department of Medical, Oral and Biotechnological Sciences, University of Chieti “G. d’Annunzio”, Chieti, Italy; Department of Systems Medicine (Neurology), Neuromuscular Disease Unit, University of Rome Tor Vergata, Rome, Italy; Department of Biomedicine and Prevention, University of Rome Tor Vergata, Rome, Italy

**Keywords:** DNAJB6, Vacuolar, Aggregation, Myopathy, TDP-43, Frontotemporal

## Abstract

**Introduction:**

Protein aggregation is a common cause of neuropathology. The protein aggregation myopathy Limb-Girdle Muscular Dystrophy 1D (LGMD1D) is caused by mutations of amino acids Phe89 or Phe93 of DNAJB6, a co-chaperone of the HSP70 anti-aggregation protein. Another DNAJB6 mutation, Pro96Arg, was found to cause a distal-onset myopathy in one family.

**Results:**

We detail the mutational, neuropathological, neurophysiological, neurological and radiological features of five new DNAJB6-myopathy families. One has the known Phe93Leu mutation and classic late-onset slowly progressive LGMD1D. Two have different mutations of Phe91 causing a variant childhood-onset severe limb-girdle myopathy. One has a Phe100Val mutation and distal-onset myopathy, unique early bulbar involvement, and a gender-modified wide age-of-onset range. The last has childhood-onset severe distal-onset myopathy and the first non-missense *DNAJB6* mutation, c.346 + 5G > A, causing a splicing defect that entirely eliminates DNAJB6’s G/F domain (ΔG/F), the domain that harbours all other mutations. Clinical and imaging examinations reveal that muscles considered uninvolved in DNAJB6-myopathy, e.g. lateral gastrocnemii, are affected in our patients with new mutations. Mutational modelling based on the known structure of the bacterial DNAJ2 protein indicates that all past and present mutated residues cluster within 15 Å in the G/F domain and all disturb the interface of this domain with the protein’s J domain that confers the interaction with HSP70.

**Conclusions:**

Our patients expand the phenotypic spectrum of DNAJB6-myopathy and allow tentative genotype-phenotype specifications. Combining with previous studies, the clinical severity spectrum is as follows: ΔG/F and Phe91 mutations, most severe; Phe100, Pro96, Phe89 mutations, intermediate; and Phe93, least severe. As it stands presently, proximal G/F domain mutations (Phe89, Phe91, Phe93) cause proximal limb-girdle myopathy, while distal G/F mutations (Pro96, Phe100) cause distal-onset myopathy. While all mutations affect the G/F–J interaction, each likely does so in different unknown extents or ways. One mutation, ΔG/F, causes its associated severe distal-onset myopathy phenotype in a clear way, through generation of a G/F domain-lacking DNAJB6 protein.

**Electronic supplementary material:**

The online version of this article (doi:10.1186/s40478-015-0224-0) contains supplementary material, which is available to authorized users.

## Introduction

Limb Girdle Muscular Dystrophy Type 1D (LGMD1D) (also called Type 1E; OMIM #603511) is, by definition, a limb girdle proximal muscle disease. Pathologically, it is characterized by sarcomeric protein aggregates, autophagic vacuolation and myofibrillar degeneration. The genetic basis of LGMD1D was discovered in 2012, namely mono-allelic mutations in the *DNAJB6* gene mapping to 7q36 [1, 2]. So far 20 families with *DNAJB6* mutations have been reported [1–5]. Of these, 19 (98 patients) have the expected limb girdle proximal muscle disease. One, an African American family, does not. Instead all affected members of this family have a distinctly distal myopathy [1].

*DNAJB6* is ubiquitously expressed, including in skeletal muscle, though with highest expression in the brain [6, 7]. DNAJB6’s best characterized function is the participation with the heat shock protein HSP70 in preventing aggregation of misfolded or otherwise aggregation-prone proteins including polyglutamine-containing huntingtin, α-synuclein, TDP-43 and Aβ42, through a process involving preferential interaction with misfolding forms of these proteins [2, 6, 8, 9]. Connection of LGMD1D to DNAJB6 raised interest in whether LGMD1D patients develop central nervous system (CNS) disease in addition to their myopathy, and in fact one patient (with a known *DNAJB6* mutation) has since been reported with pathologically confirmed frontotemporal dementia (FTD) and no mutation in any of the known FTD genes [10]. Whether this is a coincidence or other DNAJB6-myopathy patients will also develop FTD with aging remains to be seen. Recently, the autophagic vacuolation facet of LGMD1D led to the uncovering of an association between DNAJB6 and Chaperone-Assisted Selective Autophagy (CASA) components [2, 4]. A small 44 amino acid glycine/phenylalanine (G/F)-rich domain in the central portion of the protein is critical to both this association and the anti-aggregation function [2, 8, 9], and, in fact, all *DNAJB6* mutations to date (Phe89Leu, Phe89Ile, Phe93Leu/Phe93Ile and Pro96Arg) are missense mutations localized in this domain [1–5].

In 1999 a “distinctive autosomal dominant vacuolar neuromyopathy” (OMIM #601846) was described in a large Italian family with 10 affected members [11]. The disease was characterized by variable age of onset (late teens to early fifties) and severity, and by invariant initially exclusively distal leg weakness and wasting, which later spread from distal to proximal muscles of upper and lower extremities, and beyond, eventually involving most muscle groups including facial and bulbar muscles. Patients became dysphonic and dysphagic. Extraocular muscles were spared. Muscle biopsies revealed rimmed vacuoles. Genome-wide linkage analysis using microsatellite markers established linkage with an 8 cM region in chromosomal band 19p13 with a maximum two-point LOD score of 3.03. Subsequent haplotype analyses refined the locus to a 250 kb region [11, 12], but the molecular basis of this myopathy remains unknown.

Here we revisit the above family with next generation sequencing and fail to find its molecular defect in the small 250 kb region, or in the larger region of linkage in chromosome 19. Instead, we identify a previously unreported mutation in the G/F domain of the chromosome 7 *DNAJB6* gene. We also study four sporadic cases with myopathies with pathological features of LGMD1D. Three of these patients have clinically typical LGMD1D (proximal myopathy), in whom we find one known and two novel *DNAJB6* mutations. The fourth patient has a distal myopathy and a so-far unique splice-site mutation leading to complete loss of the DNAJB6 G/F domain.

Our work corrects the linkage of the distal myopathy previously assigned to chromosome 19 and identifies its cause. This family together with our patient with the splicing mutation and the published African-American family delineate the distal myopathy subphenotype of DNAJB6-myopathy and establish tentative genotype–phenotype (proximal versus distal myopathy) correlation in this disease. Finally, our work indicates that complete loss of the G/F domain function from the DNAJB6 protein underlies the distal form of the disease.

## Materials and methods

### Subjects

Patient investigations were conducted in accordance with protocols approved by the institutional review boards of the Carlo Besta Neurological Institute, Milan, the Policlinico Tor Vergata of Rome, and The Hospital for Sick Children of Toronto. Inclusion criteria for sporadic patients screened for DNAJB6 mutations (*n* = 63) were: clinical diagnosis of LGMD or distal myopathy, muscle histopathology characterized by rimmed vacuoles and/or myofibrillar abnormalities, serum CK values normal or slightly elevated and in whom other conditions such as myofibrillar myopathies or inclusion body myositis had been excluded by the appropriate investigations.

### Morphological studies

Patients III-3 and III-5 of family 1 underwent a diagnostic biopsy of the deltoid muscle. Transverse cryostat sections were routinely stained for: haematoxylin-eosin, Gomori trichrome, periodic acid-Schiff, oil red O, myofibrillar ATPase, acid phosphatase, NADH dehydrogenase, cytochrome C oxidase, succinic dehydrogenase. Immunofluorescence for phosphorylated tau-protein with anti-SMI-31 antibodies (SantaCruz Biotechnology, Santa Cruz, CA, USA) and for the autophagic protein sequestosome1 (SQSTM1, p62, monoclonal sc-28359, Santa Cruz) with anti-p62 antibodies was performed. No muscle tissue was available for additional immunochemical analyses.

On the muscle biopsies from sporadic patients (quadriceps muscle in patients 1 and 3s, biceps in patient 2s, deltoid in patient 4s), in addition to the routine histochemical and immunohistochemical analyses, immunolocalization of several other proteins was performed. Muscle cryosections were incubated with one of the following antibodies: anti-DNAJB6 (rabbit polyclonal PA5-21401, Thermo Fisher Rockford, IL, USA; 1:100), anti-LC3 (rabbit polyclonal PM036, MBL, Naka-ku, Nagoya, Japan; 1:100), anti-p62 (guinea pig polyclonal GP62-c, Progene Biotechnik, Heidelberg, Germany; 1:100;), anti-transactive response element DNA-binding protein of 43 kDa (TDP-43) (rabbit polyclonal 10782-2-AP, Proteintech Chicago, IL, USA; 1:200), anti BAG3 (mouse monoclonal sc-136493, SantaCruz; 1:50) followed by incubation in Alexa 546- or Alexa 488-conjugated goat anti-mouse, anti-rabbit, or anti-guinea pig IgG (Invitrogen Life Technologies, Carlsbad, CA, USA) as appropriate, 1:2000, for 2 h. As control, sections were either incubated with rabbit non-immune serum, or with isotype-specific non-immune IgG (Dako, Copenhagen, Denmark), or the primary antibody was omitted.

For co-localization studies muscle cryosections were incubated in a mixture of primary antibodies; either DNAJB6 (monoclonal, Sigma Aldrich, St. Louis, MO, USA; 1:100) plus TDP43, or desmin (monoclonal M0760, Dako; 1:200) plus p62, or desmin (rabbit polyclonal A0611, Dako, 1:100) plus myotilin (monoclonal NCL-MYOTILIN, Novocastra, Newcastle-upon-Tyne, UK; 1:20), or p62 plus poly-ubiquitinylated proteins (monoclonal PW8810, clone FK2 from Biomol, Enzo Life Sciences, Inc. Farmingdale, USA; 1:200), or desmin plus SMI-31 (monoclonal SMI-31R, Covance Research Products, Princeton, New Jersey, USA; 1:100) followed by washes and incubation in a mixture of appropriate fluorescent dye-conjugated secondary antibodies. Muscle sections were examined under either a Zeiss Axioplan fluorescence microscope (Carl Zeiss AG, Oberkochen, Germany) or a Leica confocal microscope equipped with hybrid and argon lasers (Leica Microsystems, Wetzlar, Germany). Muscle biopsies from X-linked myopathy with excessive autophagy (XMEA), Pompe disease (PD) and *GNE*-mutated inclusion body myopathy patients were used as positive controls.

### Western blotting

Muscle cryosections were solubilized in 25 μl lysis buffer containing 4 % SDS, 125 mM Tris–HCl pH 8.8, 40 % glycerol, and 0.5 mM PMSF; sonicated, boiled, and centrifuged at 15,000 g for 5 min. Samples of supernatant were electrophoresed on 7.5 % or 15 % SDS-PAGE and transferred onto nitrocellulose membranes. Membranes were probed with antibodies to DNAJB6 (1:500), TDP-43 (1:250), LC3 (1:500), or p62 (1:800). Vinculin (1:1000; Sigma) was used as indicator of how much muscle protein was loaded. Blocked membranes were then incubated in biotin-conjugated secondary antibody (1:2500; Jackson ImmunoResearch Laboratories, Inc., Westgrove PA, USA), followed by peroxidase-conjugated streptavidin (1:3000, Jackson ImmunoResearch) and by detection with the ECL chemiluminescence reagent (Amersham Biosciences, Buckinghamshire, UK).

### Linkage analysis

Four members of family 1 with unambiguous clinical presentation were genotyped using the Illumina HumanCoreExome Beadchip (Illumina, Inc.) following manufacturer’s instructions. Pruning of SNPs was performed with PLINK software (http://pngu.mgh.harvard.edu/purcell/plink/) [13] and the set of SNPs obtained was used for parametric linkage analysis with the MERLIN software v1.1.2 [14]. A dominant model with penetrance 0.0001, 1.0, 1.0 (wild type, hetero and homozygous alleles) was applied, calculating the LOD score for a 1 cM grid along the chromosomes at disease allele frequency of 0.0001.

### Exome sequencing

Exome sequencing was performed on two affected and one healthy relative (III:2, III:3 and III:5) as follow. Target enrichment was performed using Life Technologies Ion AmpliSeqExome Library kit according to manufacturer’s instructions. Amplified DNA was then processed for Proton sequencing. Ion PI Template OT2 200 kit v2 (Life Technologies) was used to immobilize and amplify monoclonally single stranded amplicon molecules on Ion Sphere Particles XT (ISP-XT). Sequencing was performed with the Ion Torrent Proton System using the Ion PI Sequencing 200 Kit v2 (Life Technologies). Raw signal processing, base calling, alignment and germline (diploid) variant detection was performed using Lifetech’s Torrent Server v3.6 (Life Technologies). Alignment of reads to the human genome reference hg19 (NCBI build GRCh37) was performed using Tmap 3.4.0 and single nucleotide and small insertion/deletion variant calling was performed with Torrent variant caller 3.6. The resulting variant call file (vcf) containing SNV and indels was annotated using a combination of SNPEff (http://snpeff.sourceforge.net/) and ANNOVAR [15]. Variants were filtered by screening against public single nucleotide polymorphism databases including dbSNP (http://www.ncbi.nlm.nih.gov/projects/SNP/) 1000 genomes project (http://www.1000genomes.org) and NHLBI Exome Sequencing Project (ESP) Exome Variant Server (http://evs.gs.washington.edu/EVS/). Novel SNVs were also annotated with SIFT [16] and PolyPhen-2 [17] scores to predict putative effects of variants on protein function.

### Sanger sequencing

Sanger sequencing was performed to verify and validate the variant segregating in the family and to identify mutations in sporadic cases. Genomic DNA was analysed by polymerase chain reaction (PCR) using oligonucleotide primers designed to amplify coding region, intron-exon junctions, and 1000 bp of the 5’ flanking sequence of the DNAJB6 gene (GenBank NC_000007.14). PCR products were sequenced using the BigDye Terminotor Cycle Sequencing Kit (Applied Biosystems, Foster City, CA, USA) and an ABI Prism 3100 Genetic Analyzer (Applied Biosystems). Pathogenicity predictions for variants were obtained from Mutation Tester (http://doro.charite.de/MutationTaster/), SIFT (http://sift.jcvi.org/www/SIFT_enst_submit.html), or PolyPhen-2 (http://genetics.bwh.harvard.edu/pph2/). Sanger sequencing was also performed on DNA from all the available family members of sporadic patients.

### RNA analysis

To detected alternative transcripts due to splice-site mutations, total RNA was isolated from muscle samples using TRI Reagent (Ambion, Austin, TX, USA) according to the manufacturer’s instructions, and checked for quantity and purity using a Nanodrop 2000C spectrophotometer (Thermo Scientific, Waltham, MA, USA). Aliquots of RNA (1 μg) were reverse-transcribed in the presence of 5× first strand buffer (Life Technologies, Carlsbad, CA, USA), 1 mM each deoxynucleoside triphosphate, 8 pM random hexamers, 10 μM dithiothreitol, 1 IU/μl RNAse inhibitor (Roche Molecular Biochemicals, Basel, Switzerland), and 10 IU/μl M-MLV reverse transcriptase (Life Technologies), by incubation at 37 °C for 1 h and at 95 °C for 5 min. The resulting cDNA was amplified using appropriate primers, purified from agarose gel, using QIAquick Gel Extraction kit (Qiagen, Valencia, CA, USA) according to the manufacturer’s instructions and verified by sequencing.

### Molecular modelling

Secondary structure prediction was made with PSIPRED [18] and disordered regions prediction with Robetta [19]. Both analyses suggest that the G/F domain has low structural complexity and contains a single helix that is preceded and followed by unstructured regions. The C-terminal domain of the protein is instead predicted to adopt a β structure. A search for evolutionary related homologues to DNAJB6b was performed with Phyre2 [20] and I-Tasser [21]. The initial homology model was obtained with Phyre2, using TtDNAJB2 as a template (pdbcode: 4 J80) and then energy minimized with Modeller version 9 [22]. It covers residues 2–125 of DNAJB6, which comprise the J domain, the G/F domain and its junction with the C-terminal domain. Ramachandran statistics were calculated with Procheck [23]. The geometrical quality of the model is good with 91.2 % of residues lying in the most favoured regions of the Ramachandran plot, 7.8 % in additionally allowed regions and 1 % (1 residue: Ser81) in disallowed regions. Site specific mutations were inserted with COOT [24]. All structure representations in Fig. 8 were prepared with PyMol (The PyMOL Molecular Graphics System, Schrödinger, LLC).

## Results

### Clinical and radiological findings

**Fig. 1:**
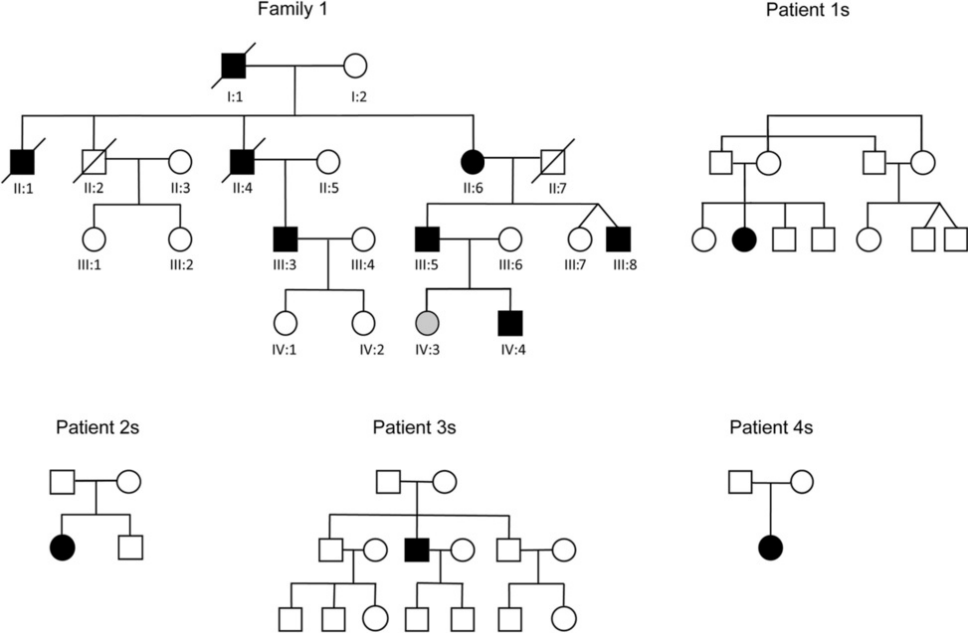
Pedigrees of family 1 and sporadic patients

Family 1 (previously thought to be linked to chromosome 19). Ten members of this family were previously considered affected. Eight of these (7 male, 1 female) have/had an unequivocal myopathy, which is inexorably progressive. Three, individuals II:2, III:1 and III:2 (see Fig. 1), in the original study [11] had a ‘particularly benign phenotype with very mild distal leg weakness and hypotrophy…’ Of these three one has since died, but the other two (III:1 and III:2) were available for re-review. With 15 years hindsight we can see that these individuals have had absolutely no progression, and are clearly not affected by the same syndrome afflicting their cousins.

Of the eight truly affected members of the family (filled symbols in family 1 in Fig. 1), five were alive and re-analyzed. Table 1 summarizes their clinical and laboratory findings. The typical pattern of muscle involvement is weakness and atrophy initiating in the distal muscles of the lower limbs and propagating to virtually all other districts, including facial and bulbar muscles, but sparing extra-ocular muscles. Foot dorsiflexor weakness was the only sign in the youngest patient (individual IV:4). Age at onset varied greatly (10 to 50 years of age). In particular, the one affected female (individual II:6) had a very late onset.

**Table 1:** Clinical, neurophysiological, pathological and radiological features of affected individuals

Patient ID	II-6	III-3	III-5	III-8	IV-4	Patient 1s	Patient 2s	Patient 3s	Patient 4s
Sex	F	M	M	M	M	F	F	M	F
Age at onset	50 y	30 y	27 y	10 y	20 y	16 y	11 y	45 y	6 y
Onset symptoms						Difficulties in climbing stairs	Impossibility to jump	Difficulties in climbing stairs	Bilateral foot drop
Age last seen	79 y	52 y	52 y	47 y	22 y	39 y	40 y (now 59 y)	57 y	39 y
Upper limb proximal weakness	severe	medium grade	severe	severe	absent	severe	severe	absent	medium grade
Upper limb distal weakness	medium grade	moderate	medium grade	severe	absent	medium grade	severe	absent	medium grade
Lower limb proximal weakness	severe	medium grade	severe	severe	absent	severe	severe	medium grade	severe
Lower limb distal weakness	severe	medium grade	severe	severe	moderate	severe	severe	medium grade	severe
Ambulant	No	Yes	Yes	No	Yes	No (lost at 30 year)	No (lost at 40 year)	Yes	No (lost at 37 years)
Facial weakness	moderate	moderate	absent	moderate	absent	absent	absent	absent	absent
Dysarthria	Yes	Yes	Yes	Yes	No	No	Yes	No	No
Dysphagia	Yes	Yes	Yes	Yes	No	No (occasional episodes in the last few months)	Yes (gastrostomy at age 58)	No	No
Dysphonia	Yes	No	Yes	Yes	No	No	No	No	No
Tongue atrophy	Yes	No	No	Yes	No	No	No	No	No
Other clinical features	Dyspnea Areflexia	Areflexia Pes cavus	Dyspnea Areflexia	Dyspnea; Contractures: thumb-wrist-elbow Areflexia	Hyporeflexia	Contractures (bilateral ankle, left knee), mild non progressive respiratory involvement	Since 57 nocturnal and daily non invasive ventilation, cough mechanical assistance,	Contractures (mild bilateral ankle)	At 17 years Achille tendons surgical correction, mild respiratory involvement
Muscle MRI	n.p.	Diffuse substitution of lower limb-girdle muscles and posterior eg muscles	Diffuse substitution of lower limb-girdle muscles and posterior leg muscles	n.p.	n.p.	Diffuse substitution of lower limb-girdle muscles and posterior leg muscles	Diffuse substitution of lower limb-girdle muscles and posterior leg muscles	Substitution of adductor magnus, femoral biceps, gastrocnemii	Thigh and leg diffuse substitution (relatively less relevant in left semitendinosus, biceps and soleus)
CK	n.p.	4x	1.5x	n.p.	n.p.	normal	1.2x	1.5x	1.1x
EMG	n.p.	Myogenic pattern; mild neurogenic changes	Myogenic pattern	Myogenic pattern	Myogenic pattern	Myogenic pattern and spontaneous activity	Myogenic pattern and spontaneous activity	Myogenic pattern	Myogenic pattern
Muscle biopsy	n.p.	Dystrophic, rimmed vacuoles	Dystrophic, rimmed vacuoles	Dystrophic, rimmed vacuoles	n.p.	Dystrophic, rimmed vacuoles	Dystrophic, rimmed vacuoles	Myopathic, rimmed vacuoles	Dystrophic, rimmed vacuoles

*n.p.* not performed

MRI of the lower limbs was performed in patient III-3 at age 52, 22 years after disease onset, and in patient III-5, again at age 52, 25 years after onset. Both were ambulant, at least indoors. MRI showed severe to almost complete fatty infiltration of lateral and medial gastrocnemii, and of the thigh muscles, in particular the vasti, adductor magnus, semitendinosus, semimembranosus and biceps femoris. Less relevant fatty infiltration was present in soleus and tibialis muscles (Additional file 1: Figure S1).

#### Sporadic patients

In three sporadic patients (1, 2 and 3s, Fig. 1) the initial symptom consisted of weakness predominantly in the muscles of the pelvic girdle, while in a fourth patient (patient 4s) the first manifestation was weakness in the lower limb distal muscles (Table 1). In patient 2s with onset at 11 years severe ventilatory failure occurred at age 57 requiring non-invasive ventilation, and severe dysphagia requiring percutaneous endoscopic gastrostomy. None of the patients had facial or tongue weakness, diplopia, ophthalmoparesis or ptosis. None had cardiac involvement, except for subaortic stenosis in patient 2s detected at age 57, and left ventricular hypertrophy on echocardiography in patient 3s at age 55 consistent with his hypertension. Family history was negative in all 4 sporadic patients.

Patients 1 and 2s underwent computed tomography (CT) examination at the age of 37 and 40, 21 and 29 years after disease onset, respectively. Both had preservation of anterior thigh muscles with the exception of vastus lateralis which was completely substituted and of soleus and medial and lateral gastrocnemius. CT of patient 3s at 52 years disclosed involvement of adductor magnus and biceps femoris along with peroneus and gastrocnemii. Patient 4s had an MRI 2 years after loss of autonomous walking (33 years after disease onset), which revealed fatty substitution of all anterior right thigh muscles and of rectus femoris and vastus medialis in the left thigh, while vastus intermedius and lateralis muscles were less severely infiltrated. Biceps femoris and semitendinosus were relatively preserved. Asymmetric involvement was also present; left soleus was less involved, while all other muscles in both legs were infiltrated.

### Morphological findings

**Fig. 2:**
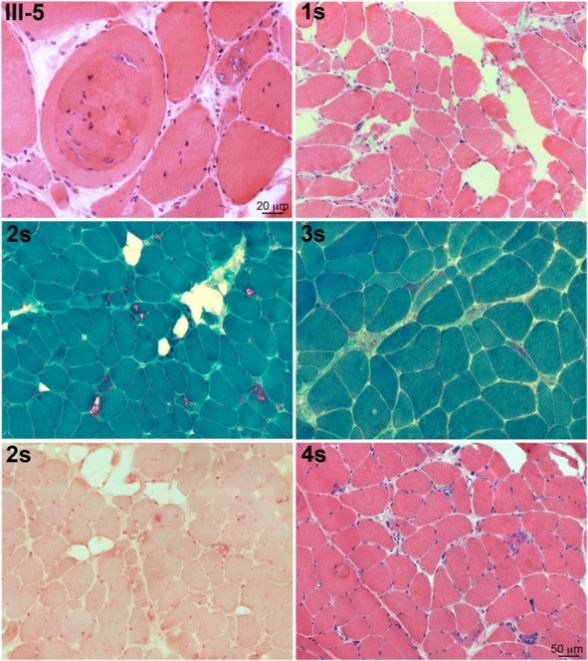
Histopathological characterization of muscle biopsies in patients III:5, and 1 to 4s, showing, by H&E and Gomori trichrome stains, rimmed vacuoles, internalized myonuclei, and variability of fiber diameters in all patient muscles; by acid phosphatase staining, increased activity surrounding vacuoles in patient 3s

**Fig. 3:**
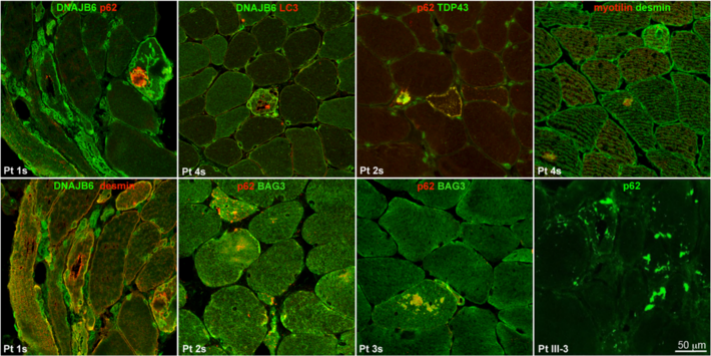
Immunolocalization of various proteins in patients 1 to 4s and III:3, showing that DNAJB6 staining is present on the rim of nuclei and in the cytoplasm of the fibers, where it co-localizes at the z discs with desmin, and on the surface and cytoplasmic inclusion of vacuolated fibers; desmin is normally expressed; myotilin is present in few inclusions in rare fibers; LC3 is present in the lumen and on the rim of vacuoles and in subsarcolemmal sarcoplasmic inclusions; p62 is present in sarcoplasmic inclusions and vacuoles with variable intracellular distribution and co-localizes with TDP43 and with BAG3

In all muscle biopsies the prevalent alterations were myopathic atrophy and hypertrophy, increased number of internalized myonuclei, necrosis and regeneration, and fibro-fatty substitution. However, the most typical finding was presence of sarcoplasmic vacuoles with basophilic rim, and some eosinophilic inclusions (Fig. 2). Immunofluorescence for phosphorylated tau was negative, whereas anti-p62 antibodies specifically decorated sarcoplasmic inclusions with various intracellular distribution (Fig. 3).

Both in family 1 and in the sporadic patients, in addition to rimmed vacuoles in a variable number of fibers, there was also extreme variability of fiber diameters, focally reduced activity of oxidative enzyme staining and increased acid phosphatase activity surrounding the vacuoles (Fig. 2). Limitation of muscle tissue from family 1 did not allow additional studies, which were possible in the sporadic cases. In the latter, DNAJB6, by immunofluorescence, was expressed at the peripheral rim of most nuclei, in the cytoplasm of fibers, localized at the Z discs (when observed in longitudinal fibers), and on the surface and within inclusions of vacuolated fibers (Fig. 3); LC3 positivity was detected in the lumen and on the rim of vacuoles and in subsarcolemmal sarcoplasmic inclusions (Fig. 3), p62 was strongly expressed as aggregates in and around vacuoles, as dots in the cytoplasm of a few fibers and as a rim delimiting the surface of rare fibers (Fig. 3). TDP-43 was similarly expressed and co-localized with the p62 signal (Fig. 3), and, in addition, was expressed in nuclei. Desmin was normally expressed in the intermyofibrillar network, myotilin was observed in several positive sarcoplasmic aggregates in a few fibers (Fig. 3), and SMI-31 was expressed within few vacuoles (not shown). BAG3 expression was increased in the cytoplasm of vacuolated and atrophic angulated fibers and within aggregates, often co-localizing with p62 (Fig. 3).

### Western blot analysis

**Fig. 4:**
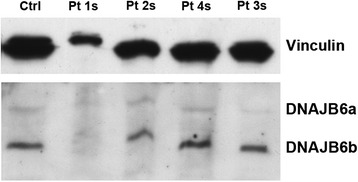
Western blot of DNAJB6 protein in muscle homogenates from sporadic patients compared to control showing both DNAJB6 isoforms

By Western blotting both DNAJB6 long and short isoforms were expressed in patient muscle biopsies except in patient 1s in whom all muscle proteins were extremely reduced (Fig. 4). We did not observe obvious differences in expression of LC3, p62 or TDP-43 compared to control muscles (not shown), probably because the technique fails to reveal changes that are present in a small number of fibers.

### Genetic analysis

**Fig. 5:**
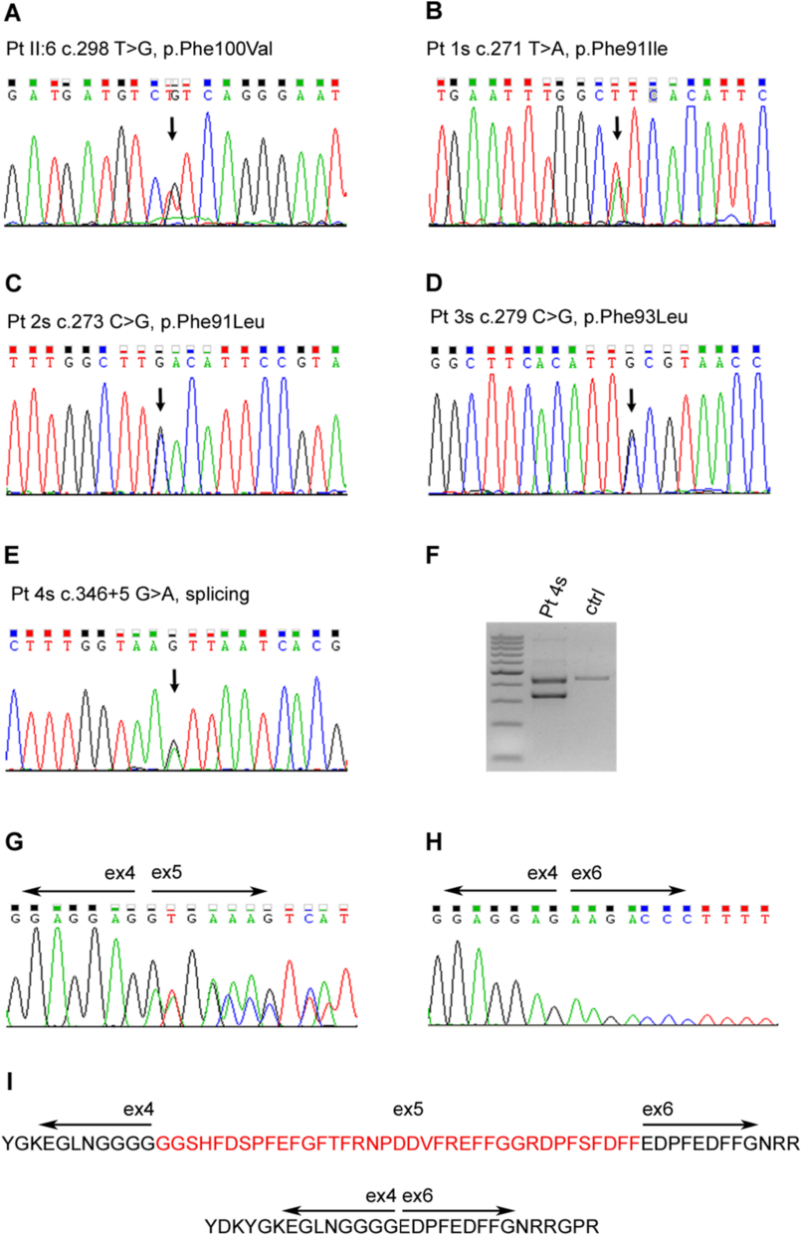
**a**–**e**) Electropherograms showing the mutations in genomic DNA from patients II:6 and patients 1 to 4s. **f** Agarose gel showing the alternative transcript in patient 4s’ cDNA compared to control, and (**g**–**i**) sequences confirming skipping of exon 5, which encodes the G/F domain, in the smaller cDNA band and loss of amino acid 79_115 coded by exon 5

In family 1, SNP based linkage analysis identified 18 genomic regions with LOD scores ≥ 1. Among 22 meaningful variants found in these regions by exome sequencing after application of filters, a heterozygous missense variant c.298 T > G causing a Phe100Val change in the chromosome 7 *DNAJB6* gene (NM_058246.3) was noted (Fig. 5). Sanger sequencing confirmed this variant in heterozygous state in all affected members. The variant is novel and not reported in variant databases such as 1000 Genomes, Exome Server Project (ESP), dbSNP142 and the EXAC browser (http://exac.broadinstitute.org/). It falls in the highly conserved region of the DNAJB6 G/F domain. Predictive software tools such as SIFT, PolyPhen-2 and Mutation Taster, suggested a strong deleterious effect on the protein. Finally the phenylalanine in this position is conserved through evolution from *xenopus* to human. The variant was absent from all healthy relatives, except one, individual IV:3 (age 30 years). This female individual carries the mutation but is not yet clinically affected. As mentioned, her grandmother, individual II:6 and the only affected female in the family, was not affected until age 50. Individuals III:1 and III:2 do not carry the mutation. Of note, no variants were present in the 8 cM chromosome 19 region delimited by the original study [11], i.e. between D19S209 to D19S177. Moreover no known vacuolar myopathy genes including *GNE, DES, MTR3, VCP, MYH2, FLNC, TCAP, ANO5* and *NEB* had any variants in the exome results. *TTN* did show three common previously reported benign variants.

In the sporadic cases we detected by Sanger sequencing one previously reported missense mutation [1, 2], c.279C > G p.Phe93Leu in patient 3s. Patients 1 and 2s had novel mutations, c.271 T > A p.Phe91Ile, and c.273C > G p.Phe91Leu, respectively, affecting the same amino acid. In patient 4s we found an intronic change, c.346 + 5G > A, in the consensus splice site sequence. RNA analysis in this patient revealed a normal band and a band of lower molecular weight that, by sequencing, was missing the entire exon 5, which encodes the G/F domain (Fig. 5). This mutation is termed ΔG/F in the remaining text. All the mutations were checked also in available family members. None had the corresponding variants, i.e. all were de novo mutations. Other vacuolar myopathy related genes previously ruled out in these sporadic patients by Sanger sequencing were: *GNE* and *ANO5* for patient 1s, *GNE* for 2s, *GNE, ANO5* and *MYH7* for 3s and *GNE* for 4s.

### Mutation molecular modelling

DNAJB6 belongs to the Type II subfamily of Hsp40 proteins, whose structure can be roughly divided into three functional domains: the conserved N-terminal J domain, the G/F domain, and a variable C-terminal domain [25]. The above results, combined with all previous published cases [1–5], reveal that mutations of Phe89, Phe91 and Phe93 are associated with proximal-onset myopathy, while mutations of Pro96 (Pro96Arg, the African-American family mentioned above) and Phe100, and ΔG/F, result in distal-onset myopathy.

**Fig. 6:**
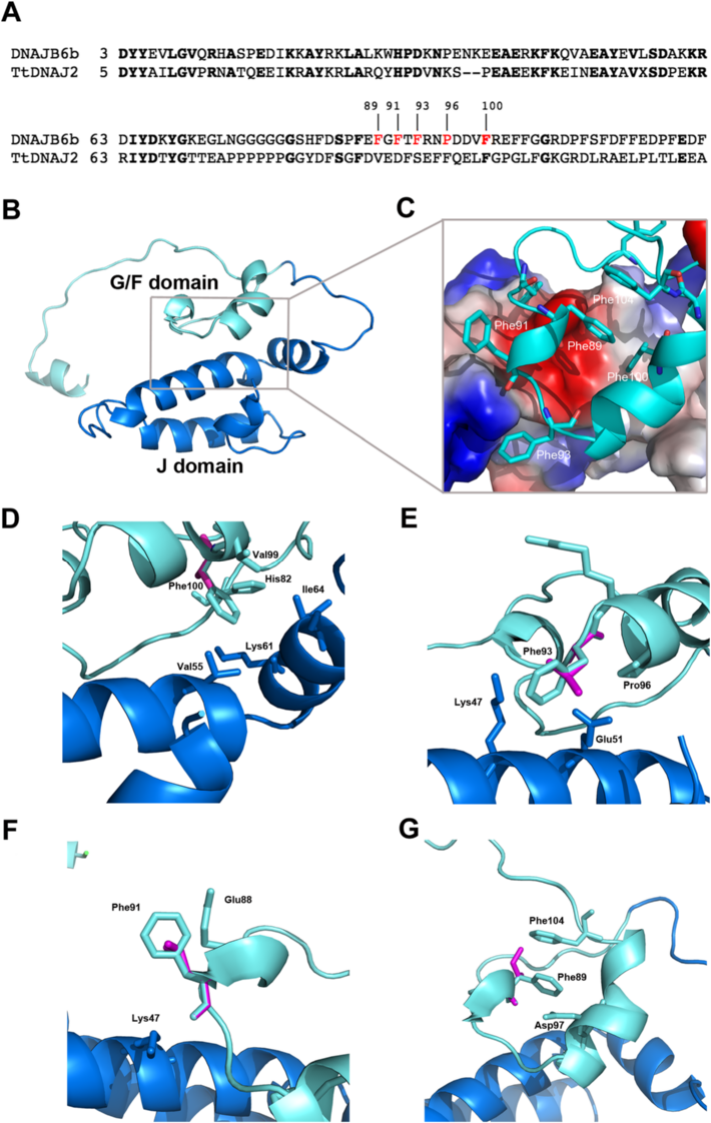
Putative structure of DNAJB6b J and G/F domains obtained through homology modelling using the structure of TtDNAJ2 (pdbcode: 4 J80) as template. **a** Alignment of DNAJB6b and TtDNAJ2 highlighting the J and G/F domains. Identical residues are shown in bold, residues found mutated in patients are highlighted in red. **b** Ribbon representation of the homology model of DNAJB6. **c** A detail of the interface between J and G/F domains. The J domain is represented in surface and colored according to its electrostatic properties (blue for positive and red for negative charge, respectively) while the G/F domain is shown in cyan ribbon. Phenylalanine residues found at the interface are shown in sticks. **d** Detailed view of the interactions played by Phe100. Its corresponding valine residue found in patients is shown in magenta. The J domain is represented in blue while the G/F domain is represented in cyan. Residues close to Phe100 are also shown in sticks; Phe100 is predicted to establish interactions with both J domain residues (Val55, Lys61 and Ile64) and G/F domain residues (His82 and Val99). **e** Detailed view of the Phe93Leu mutation; Phe93 is predicted to establish a cation-π interation with J domain residue Lys47 and hydrophobic interactions with G/F domain Pro96. Pro96 faces the J domain helix III and its mutation to arginine is linked to distal-onset phenotype. **f** Detailed view of the Phe91Leu mutation; interacting residues are also shown. **g** Detailed view of the Phe89Ile mutation. Phe89 does not directly contact the J domain; rather it protrudes inside the G/F domain spiral where it is predicted to interact with Phe104. Color codes in (**e**, **f** and **g**) panels are the same as in (**g**)

To gain possible insights into mutation-dependent phenotypic differences we obtained a homology model for DNAJB6. We interrogated the Phyre2 [20] and I-Tasser [21] servers for structural homologues and both identified the *Thermus thermofilus* DNAJ2 protein (TtDNAJ2; pdb code: 4 J80) [26] as the closest DNAJB6 homologue of known structure, with 100 % confidence in the evolutionary relationship between the two proteins (Phyre2). Fig. 6a shows the alignment of DNAJB6 and TtDNAJ2 comprising the J and G/F domains and the junction between the latter and the C-terminal domain. The homology model was thus built, on the basis of the Phyre2 alignment, with TtDNAJ2 as a single template, and then energy minimized using Modeller [22] (Fig. 6b). We did not model the C-terminal domain because no clear consensus could be derived in this region by the alignment programs.

The G/F domain is predicted to be composed of an α-helix and flanking loops. Pro96 and Phe100 are in the α-helix, while Phe89, Phe91 and Phe 93 are in the upstream loop. In the model, the G/F domain folds as a spiral onto the J domain with which it makes widespread contact, mediated through phenylalanine residues, including Phe91, Phe93 and Phe100 (Fig. 6c). Conservative replacements of these phenylalanines are predicted by the model to disrupt or weaken the G/F – J domain interactions (Fig. 6d, e and f). Replacement of Pro96 with arginine is likewise predicted to destabilize the G/F – J interface because of arginine’s positive charge and cumbersome side chain compared to proline’s (Fig. 6e). Phe89 is predicted to have a somewhat different impact as it does not directly interact with the J domain. Rather, its side chain protrudes inside the spiral formed by the G/F domain where it makes hydrophobic interactions, among which a stacking interaction with Phe104 which if lost would destabilize the G/F spiral (Fig. 6g). Overall, the homology model suggests that all mutated residues cluster in the same area of the G/F domain. The model also suggests that mutations, even when conservative, might all disturb the J-G/F interdomain interactions or destabilize the G/F domain, or both.

## Discussion

*Proximal* versus *distal onset myopathy* – *DNAJB6* mutations established causation in LGMD1D [1, 2]. In these original and subsequent publications all mutations in all cases with limb girdle myopathy were missense and all were in the encoded protein’s G/F domain, specifically in the codons of amino acids 89 and 93 [1–5]. In one of the two original reports, one of the families (mutation Pro96Arg) did not have a limb girdle myopathy but a distal myopathy [1]. In the present report, two of our sporadic cases (1 and 2s) have novel missense mutations affecting the codon of amino acid 91. Our third sporadic case (3s) has a known mutation of amino acid 93. All three have limb girdle myopathy. On the other hand, affected members of our family 1 have a mutation in amino acid 100 and manifest a distal-onset myopathy. As such, as of now, all published cases with mutations in amino acid 93 and proximal have a proximal, limb girdle, myopathy, while cases affecting the more distal amino acids 96 and 100 have a distal myopathy. Finally, our sporadic case with ΔG/F has a distal myopathy, suggesting that the clinical effect of mutations in the distal amino acids 96 and 100 is more similar, at least in regards to the order of clinical progression, to loss of the G/F domain.

*Sex effect* – In our family 1, six of seven affected individuals are male and the one female (II:6) has a much later onset (Fig. 1 and Table 1). In one sibling pair in the family (IV:4 and IV:3), the male (IV:4) is affected at age 22, whereas the female (IV:3), who also carries the mutation, is still unaffected at 30. We counted the affected males and females in the published literature [1–5], including ours, and find that males outnumber females 71 to 46 (3:2). These results suggest a gender modifier in DNAJB6-myopathy due to the Phe100Val mutation. In our other, sporadic, patients we could not perform a similar analysis and the generalizability of the sex effect awaits study of multiplex families with the other *DNAJB6* mutations.

*Age of onset and severity* – Our patient with the previously known Phe93Leu had his onset at age 45, progressed slowly and is still ambulatory at age 57 (Table 1), consistent with the classic adult onset LGMD1D phenotype and all 49 previously reported cases with this mutation [1, 2, 4].

The other previously published mutations in LGMD1D were both in amino acid 89 (Phe89Ile and Phe89Leu), and with these mutations onsets in both adulthood and childhood (as young as age 8) were reported, with a disease course somewhat more rapid than the descriptions of Phe93Leu/Phe93Ileu patients, indicating that Phe89Ile/Phe89Leu are somewhat more severe than Phe93Leu/Phe93Ileu.

Both our cases with the novel Phe91Ile and Phe91Leu mutations have childhood onset disease (16 and 11 years-old respectively), and one of them (patient 2s; Phe91Leu) has had a relatively severe course. She lost ambulation at age 40, had major respiratory difficulties since age 52, requiring daily nocturnal non-invasive ventilation since age 57, and severe swallowing difficulty with consequent marked weight loss necessitating gastric feeding at age 58. It appears that patient 1s (Phe91Ile) is following a similar severe course; she is already non-ambulant at age 30 (Table 1).

Our patients with Phe100Val mutation and distal-onset myopathy have a range of ages of onset (10 to 50) and severity, as mentioned. Additionally, they have dysphagia and early bulbar involvement with dysarthria, dysphonia and tongue atrophy, features not seen with any other mutations. The only other cases with a missense mutation and distal-onset myopathy (Pro96Arg) had a range of age of onset from 18 to 35.

Our patient with ΔG/F and distal-onset myopathy (4s) has the earliest onset DNAJB6 myopathy phenotype reported to date (age 6). Her progression appears to be following the severe course of the Phe91 cases. She lost ambulation at 37 years and at 39 years has commenced to have respiratory difficulties (Table 1).

## Conclusions

In summary, it appears that the range of severity in DNAJB6 myopathy in presently known mutations is as follows: ΔG/F and Phe91 mutations, most severe – Phe100Val, Pro96Arg and Phe89 mutations, intermediate severity – Phe93 mutations, least severe. This obviously awaits confirmation in larger numbers of patients.

*Skeletal muscle imaging* – Following the reporting of the first LGMD1D *DNAJB6* mutations, Udd and colleagues published a detailed muscle MRI study of the disease. Only their European (Finnish and Italian) cases were included, as their American families were older cases without adequate imaging available. As such, all the patients studied had the Phe93Leu mutation. The authors reported the radiological features of these cases in both early and late phases of the disease, disclosing a pathognomonic pattern of muscle involvement for this mutation - early stages: ‘soleus, adductor magnus, semimembranosus and biceps femoris muscles followed by medial gastrocnemius, adductor longus and later by vasti muscles of the quadriceps. Rectus femoris, lateral gastrocnemius, sartorius, gracilis and the anterolateral group of the lower leg muscles were spared until late senescence [27]. In our present cases, the Phe93Leu patient fits the above pattern. To our knowledge one other study reporting MRI findings has been published, namely several Japanese patients with Phe93Ile, with findings comparable to the Phe93Leu cases [4]. In our current other patients with more severe, or with distal onset disease, the recti, sartorii, gracilis and anterolateral groups did show relative sparing, but the lateral gastrocnemii were seriously involved in all patients including in less advanced stages of disease.

DNAJB6 *mutations explain 6.4 % of our collection of genetically undefined vacuolar and myofibrillar myopathies* – *DNAJB6* mutations were present in 4 of 63 of our patients followed at the Besta Neurological institute with LGMD or distal phenotype, muscle histopathology characterized by vacuolation and/or myofibrillar abnormalities, and no mutations in the known genes associated with these pathologies. Additional genes therefore remain to be identified in these myopathies.

*Functional insights* – Evidence strongly suggests that DNAJB6-myopathy mutations are gain-of-deleterious-function mutations. Protein mutated at Phe89, Phe93 and Pro96, or lacking the G/F domain (ΔG/F), introduced into yeast and zebrafish models, in amounts equimolar to wild-type their counterpart, have toxic consequences consistent with the disease pathology [2, 9]. Canonically, J domain containing proteins interact with target misfolding proteins through their C-termini and then with HSP70 through the J domain, which initiates processes of either restabilizing the target or escorting it to degradation [28]. While it is unclear whether DNAJB6 follows this precise rule, it is abundantly clear that the protein prevents aggregation of varied misfolding-prone proteins [6, 8, 9]. The DNAJB6 G/F spiral folds upon and makes what appear to be crucial contacts with the J domain through its phenylalanines. All three previously known *DNAJB6* mutations (of Phe89, Phe93 and Pro96) and mutations reported here (of Phe91 and Phe100 and ΔG/F) are strongly predicted to act, at least in part, through disturbing these contacts. Our immunohistochemical results, showing cytoplasmic protein aggregates in the cytoplasm of many fibers in all patients, failed to reveal a particular relation between histopathological aspects and specific mutations [1, 2].

The clinical outcomes of the mutations while similar (DNAJB6 myopathy) are not identical and not merely in severity, but also in particularities of skeletal muscles involved, i.e. lateral gastrocnemii with Phe91, Phe100 and ΔG/F, and early bulbar involvement with Phe100. This suggests a highly intricate and selective activity of the protein at the G/F – J interface. A glimpse into this has already been obtained through the remarkable observation that at least the previously known mutations affect the processing of particular, and not all, conformers of target misfolding proteins [9]. Understanding the precise molecular mechanisms through which DNAJB6 acts to process or stabilize misfolding proteins are of keen interest for this and other protein aggregation myopathies and for common neurological diseases, including Alzheimer’s disease and frontotemporal dementias. The current, and potentially future, mutations in DNAJB6-myopathy patients will be crucial guides in obtaining this knowledge.

### Consent

Informed consent was obtained from the patients in accordance with institutional review board guidelines of the Besta Institute of Milan, the Policlinico Tor Vergata of Rome, and The Hospital for Sick Children of Toronto.

## Additional file

Additional file 1: Figure S1. Muscle MRI (T1W/TR) of patients III-3 (lower panel) and III-5 (upper panel). At mid-thigh level, a severe and diffuse fibro-fatty substitution of all muscles is evident (Pt. III-5). At mid-leg level, a severe substitution of posterior leg muscles is shown, whereas muscles of the anterior compartment are relatively spared (Pt. III-3). MRI in patient 4s at mid-thigh level shows relative sparing of biceps femoris, and, at mid-leg level, that the left soleus is less affected than other muscles.
