# Supplementary figures and images for: Complete loss of the DNAJB6 G/F domain and novel missense mutations cause distal-onset DNAJB6 myopathy
Source: Acta Neuropathol Commun. 2015 Jul 25;3:44. doi: 10.1186/s40478-015-0224-0 (PMC4513909; doi:10.1186/s40478-015-0224-0)

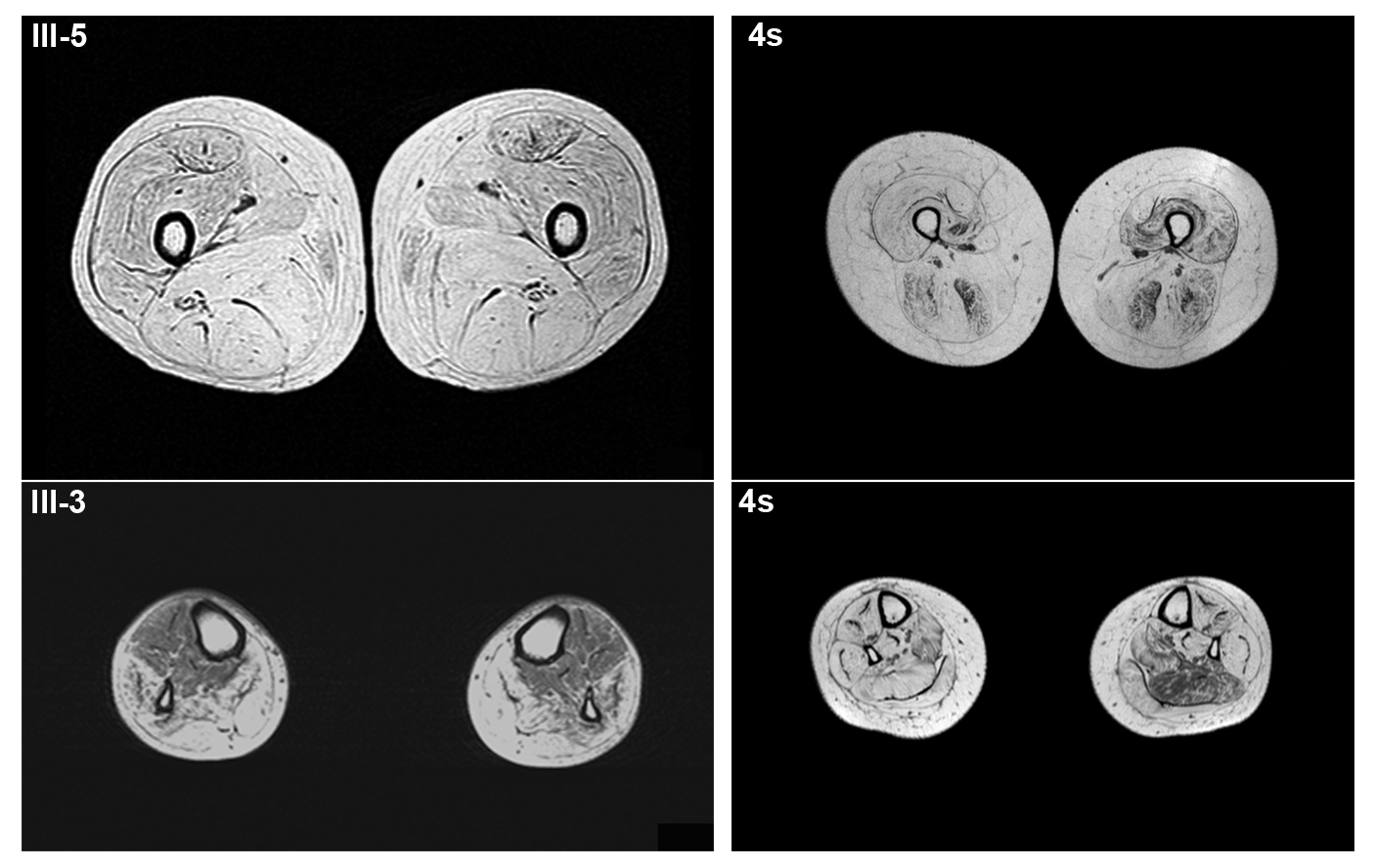

Supplement: Additional file 1: Figure S1. — Muscle MRI (T1W/TR) of patients III-3 (lower panel) and III-5 (upper panel). At mid-thigh level, a severe and diffuse fibro-fatty substitution of all muscles is evident (Pt. III-5). At mid-leg level, a severe substitution of posterior leg muscles is shown, whereas muscles of the anterior compartment are relatively spared (Pt. III-3). MRI in patient 4s at mid-thigh level shows relative sparing of biceps femoris, and, at mid-leg level, that the left soleus is less affected than other muscles. [file 40478_2015_224_MOESM1_ESM.tif]
